# The association of exhaled nitric oxide with air pollutants in young infants of asthmatic mothers

**DOI:** 10.1186/s12940-023-01030-6

**Published:** 2023-12-05

**Authors:** Elizabeth Percival, Adam M. Collison, Carla Rebeca da Silva Sena, Ediane De Queiroz Andrade, Patricia De Gouveia Belinelo, Gabriela Martins Costa Gomes, Christopher Oldmeadow, Vanessa E. Murphy, Peter G. Gibson, Wilfried Karmaus, Joerg Mattes

**Affiliations:** 1 Asthma & Breathing Research Centre, Hunter Medical Research Institute, University of Newcastle, Newcastle, NSW Australia; 2 Hunter Medical Research Institute, New Lambton Heights, New South Wales, Australia; 3 Department of Respiratory and Sleep Medicine, John Hunter Hospital, Newcastle, NSW Australia; 4 Division of Epidemiology, School of Public Health, and Environmental Health Science, University of Memphis, BiostatisticsMemphis, TN 38152 USA; 5 Department of Paediatric Respiratory & Sleep Medicine, John Hunter Children’s Hospital, Newcastle, NSW Australia

**Keywords:** Exhaled nitric oxide (eNO), Infant, Air pollution, Breathing for Life Trial, Season

## Abstract

**Background:**

Exhaled nitric oxide is a marker of airway inflammation. Air pollution induces airway inflammation and oxidative stress. Little is known about the impact of air pollution on exhaled nitric oxide in young infants.

**Methods:**

The Breathing for Life Trial recruited pregnant women with asthma into a randomised controlled trial comparing usual clinical care versus inflammometry-guided asthma management in pregnancy. Four hundred fifty-seven infants from the Breathing for Life Trial birth cohort were assessed at six weeks of age. Exhaled nitric oxide was measured in unsedated, sleeping infants. Its association with local mean 24-h and mean seven-day concentrations of ozone, nitric oxide, nitrogen dioxide, carbon monoxide, sulfur dioxide, ammonia, particulate matter less than 10 μm (PM10) and less than 2.5 μm (PM2.5) in diameter was investigated. The air pollutant data were sourced from local monitoring sites of the New South Wales Air Quality Monitoring Network. The association was assessed using a ‘least absolute shrinkage and selection operator’ (LASSO) approach, multivariable regression and Spearman’s rank correlation.

**Results:**

A seasonal variation was evident with higher median exhaled nitric oxide levels (13.6 ppb) in warmer months and lower median exhaled nitric oxide levels (11.0 ppb) in cooler months, *P* = 0.008. LASSO identified positive associations for exhaled nitric oxide with 24-h mean ammonia, seven-day mean ammonia, seven-day mean PM10, seven-day mean PM2.5, and seven-day mean ozone; and negative associations for eNO with seven-day mean carbon monoxide, 24-h mean nitric oxide and 24-h mean sulfur dioxide, with an R-square of 0.25 for the penalized coefficients. These coefficients selected by LASSO (and confounders) were entered in multivariable regression. The achieved R-square was 0.27.

**Conclusion:**

In this cohort of young infants of asthmatic mothers, exhaled nitric oxide showed seasonal variation and an association with local air pollution concentrations.

**Supplementary Information:**

The online version contains supplementary material available at 10.1186/s12940-023-01030-6.

## Background

Exhaled nitric oxide (eNO) is a non-invasive marker of eosinophilic airway inflammation clinically used in adults and school-aged children with asthma [1, 2].

Nitric oxide (NO) is generated by the oxidation of L-arginine by nitric oxide synthase (NOS) by epithelial, inflammatory and vascular endothelial cells in the respiratory tract [3]. There are three different isoforms of NOS, each with predominantly proinflammatory (inducible NOS or NOS2), or physiological (neural NOS or NOS1 and endothelial NOS or NOS3) effects [3]. In the lung, NOS3 is responsible for many physiological effects including: lung development, airway smooth muscle relaxation, protection against bronchoconstriction stimuli and ciliary motility [4], while NOS2 has been shown to have inflammatory and innate immune defence effects by regulating the function and activity of T cells, antigen presenting cells, mast cells, neutrophils and NK cells [5, 6].

In addition to asthma, elevated eNO has been reported in other inflammatory disorders in infants: established bronchopulmonary dysplasia [7], eczema [8] and airway hyperresponsiveness in infants with recurrent lower respiratory tract symptoms [9]. Contrasting this, we have recently shown that lower eNO at six weeks of age is associated with higher rates of bronchiolitis and wheeze in the first year of life [10], and decreased eNO has been described in infants with cystic fibrosis [11], respiratory syncytial virus bronchiolitis [12], wheezy bronchitis [13], and rhinorrhoea [14] making the role of eNO in respiratory disorders manifesting in early life more cryptic.

eNO has been shown to be lower in infants with prenatal tobacco exposure (PTE) [15], in infants with nonatopic mothers and PTE [16] and in infants exposed to combined pre- and postnatal tobacco smoke [17]. Diverging from this is that infants born to mothers who ceased smoking during pregnancy and resumed after the infant’s birth have higher eNO [16], suggesting a different response for those exposed to tobacco smoke solely in the ex-utero environment. Whether this difference could be related to timing of exposure (recent, acute exposure compared with more prolonged exposure in-utero and after birth), mechanism of exposure (placental circulation compared with direct airway epithelial exposure) or another mechanism entirely is not established.

The Bern Basel Infant Lung Development (BILD) cohort study [18] reported that eNO was associated with prenatal exposure to nitrogen dioxide (NO_2_), but found no association with cumulative postnatal particulate matter < 10 μm in diameter (PM10), NO_2_ and ozone (O_3_) exposure and eNO at five weeks of age.

While there is no published data on the effect of recent (seven days or less) exposure to air pollutants on eNO in infants, elevated eNO in children has been associated with recent exposure to environmental air pollutants including NO_2_, O_3_, carbon monoxide (CO), benzene, PM10 and particulate matter < 2.5 μm (PM2.5) [19–21].

Infants born to mothers with asthma in pregnancy have higher rates of respiratory complications after birth [22] and higher risk of developing asthma [23], making this group of infants particularly vulnerable to further insults to their respiratory system. We therefore sought to investigate the relationship between eNO and recent exposure to postnatal environmental air pollutants in young infants from a birth cohort born to mothers with asthma in pregnancy.

## Methods

### Study population

Infants were recruited as part of a prospective nested cohort study from the Breathing for Life Trial (BLT). As previously described [24] BLT recruited pregnant women, aged 18 years or older with physician-diagnosed asthma, self-reported symptoms of asthma or use of asthma therapy (beta-2 agonist or inhaled corticosteroids) to receive randomized asthma management intervention during their pregnancy. Women were recruited between 12–23 weeks’ gestation and randomized to either usual asthma management or fractional exhaled nitric oxide (FENO)-based management. Sociodemographic and maternal smoking status were collected by interviewer-administered questionnaire at first maternal study visit in BLT. Pregnancy, delivery and neonatal outcome data were obtained from medical records [25].

After birth infants were clinically assessed in Newcastle, Australia at six weeks corrected gestational age. Inclusion criteria for eNO measurement was no apparent major birth defects or perinatal disease that would preclude performing unsedated infant lung function testing and that the infant had not had a respiratory illness in the two weeks prior to testing.

### Ethics and consent

The Hunter New England Human Research Ethics Committee Human Research Ethics Committee (ref no: 12/10/17/3.04) and University of Newcastle Human Research Ethics Committee (ref no: H-2012–0422) approved the study. Informed written consent was obtained from all parents or guardians prior to entry into the study.

### Clinical assessment

One day prior to the clinical assessment, a researcher contacted the family to ensure that the infant was well and did not have any respiratory symptoms (blocked nose, runny nose, wheezing, cough or any other respiratory symptoms) in the two weeks prior to the appointment. Infants were assessed for respiratory health by questionnaire, eNO measurement, physical examination and growth parameters (length, weight, head circumference) by a paediatrician or paediatric nurse between March 2015 and December 2019.

### eNO measurement

eNO measurement was performed during unsedated, behaviourally defined quiet sleep. Infants were tested in the supine position, with infant sized masks (sizes 0, 0/1 and 1; Homedica AG, Huenenberg, Switzerland), according to the European Respiratory Society (ERS)/American Thoracic Society (ATS) standards for infant eNO measurement [26], with mask dead-space corrected for during analysis. Flow was measured with an ultrasonic flow meter (Spiroson®; EcoMedics AG, Duernten, Switzerland). Flow calibration was performed daily. eNO was measured online with a rapid response chemiluminescence analyzer (CLD88; EcoMedics AG, Duernten, Switzerland) in the range 0–100 ppb and an error rate of ± 1 ppb. At least three trials with a coefficient of variance (CV) within 10 percent were measured. As previously validated by Hall et al., [15] the time-based third quartile eNO value was chosen because intraindividual variability of breaths was lowest. The chemiluminescence analyzer was serviced annually (including calibration to reference gas) as per the manufacturer guide. To ensure consistency of analysis [15], only the first 100 tidal breaths were analysed (Spiroware 2.0 EcoMedics AG, Duernten, Switzerland). Considering a mean respiratory rate of 44 breaths per minute in this cohort, data collection of 100 breaths would take at least two to three minutes. As eNO is flow dependent and to account for interindividual variability in flow, eNO values were interpolated to a flow rate of 50 mL/s (eNO50) using GraphPad Prism version 9.1 (GraphPad Software, San Diego, California USA). Ambient nitric oxide (NO) was measured before each assessment. When ambient NO exceeded 5 ppb, NO-free air for inhalation during testing was supplied via a DENOX 88 module, (EcoMedics AG, Duernten, Switzerland) connected to the chemiluminescence analyzer. When ambient NO exceeded 5 ppb and the module was not functional, the eNO results were excluded from the analysis.

### Air pollution and weather

Mean hourly concentrations (in micrograms per cubic metre at 25 degrees Celsius, except CO in milligrams per cubic meter) of O_3_, NO, NO_2_, CO, Sulphur dioxide (SO_2_), Ammonia (NH_3_), PM10 and PM2.5 were sourced from the New South Wales (NSW) Air Quality Monitoring Network [27]. Data were obtained from the Newcastle monitoring site (6.4 km from the testing facility), except NH_3_ which was only available from the Stockton monitoring site 8.9 km away from testing facility. The monitoring stations are accredited by the National Association of Testing Authorities, recording real-time air pollutant concentrations [28]. As infants were typically assessed at 12 noon (± 2 h), the mean concentration for the 24 h to 12 pm local time (Australian Eastern Standard Time or Australian Eastern Daylight Time when applicable) was calculated from the hourly data. Since there is evidence for lagged effect of air pollutants [20, 21], seven-day mean values were also calculated for each air pollutant in the same way.

Maximum daily temperature data were achieved from the Australian Government’s Bureau of Meteorology [29] for the University of Newcastle site (3.8 km from testing facility). Mean daily temperature, mean daily humidity and 9am humidity were all sourced from the New South Wales (NSW) Air Quality Monitoring Network [27].

### Statistics

Stata 16.1 (StataCorp, College Station, Texas, USA) and Prism 9.1 for macOS (GraphPad Software, La Jolla California, USA) were used for statistical analysis and graphical presentation. Heat maps were created in Microsoft Excel for Mac 16.43 (Microsoft corporation, Redmond, Washington, USA). Participant clinical features, eNO, weather and air pollutants are presented as medians with minimum and maximum values for continuous variables and frequency with percentages for categorical variables. Differences between infant groups were tested with Mann–Whitney two-tailed test for continuous variables and Fisher’s exact test for categorical variables. *P* values < 0.05 were considered statistically significant.

eNO50 concentrations were right skewed with the mean greater than the median. To achieve a near normal distribution, these values were transformed to their square root (eNOSqR).

To test for seasonality of eNO related to mean daily temperature, the months of the year were categorized by mean daily temperatures above or below 20 degrees Celsius, with November to March being above and April to October being less than 20 degrees. Differences between the groups (mean daily temperature above or below 20 degrees Celsius) were tested with the Mann–Whitney two-tailed test for non-normally distributed data. Due to increasing seasonal variation in eNO results, month of test was parameterized with a sine model “sinterm” (assumed value of zero at June, peak in March, and trough in September with increasing amplitude for each year) for inclusion in subsequent regression analysis of eNO. To test for seasonal variation in air pollutants, month of test was parameterized with a cosine model (assumed value of zero in March and September, trough in June, and peak in December).

A ‘least absolute shrinkage and selection operator’ (LASSO) approach was used to reduce the dimensionality of the air pollution data and estimate model coefficients. Lambda was determined based on tenfold cross-validation. Seventeen variables of interest were included in the LASSO for eNO: eight 24-h mean to 12 pm air pollutants (NO, NO_2_, O_3_, SO_2_, CO, PM10, PM2.5, NH_3_), eight seven-day mean air pollutants (NO, NO_2_, O_3_, SO_2_, CO, PM10, PM2.5, NH_3_) and sinterm (to account for seasonal variation); plus seven known or possible confounders for eNO: male sex, prematurity, birth order, birth weight, chronological age at test, maternal smoking in pregnancy and exclusive breastfeeding up to the six-week visit [15, 30–33].

Spearman’s rank correlation coefficients were calculated for eNO, sinterm and seven-day mean air pollutants, as well as by season with a heat map generated for the same variables.

As there is limited data on the association between eNO and recent (seven days or less) postnatal environmental air pollution exposure in young infants, a power calculation could not be performed prior to the study.

## Results

**Fig. 1:**
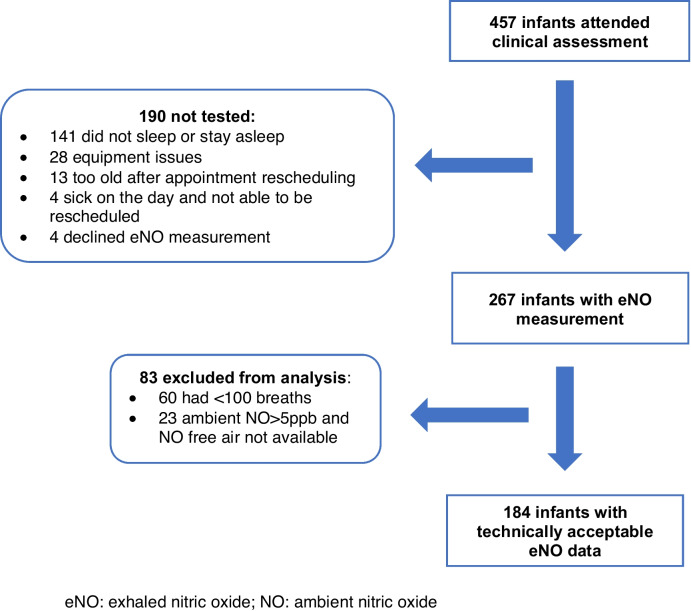
eNO: exhaled nitric oxide; NO: ambient nitric oxide

Four hundred fifty-seven infants attended an appointment at six weeks corrected gestational age for clinical review, and eNO measurement. Two hundred sixty-seven (58% of 457) attempted eNO measurement (Fig. 1 – Flowchart for participation in the eNO testing). Measurements were precluded in the remaining due to infants not sleeping (or staying asleep) and equipment issues. Technically acceptable eNO measurements were obtained in 184 infants (69% of 267). Reasons for not obtaining technically acceptable data included infant not achieving 100 breaths with CV less than 10% and ambient NO levels greater than 5 ppb, when NO free air was not available due to equipment issues.

Baseline demographic, anthropometric and eNO results are shown in Table 1, with no significant differences between those who had a valid eNO measurement and those who did not. The groups were similar regarding sex (50–57% male), prematurity (~ 10%), mode of delivery (born via caesarean section ~ 35%) and prenatal tobacco exposure (10%).

**Table 1:** Demographic, anthropometric, and eNO

Demographic data	Valid eNO group (*n* = 184), *n (%)*	No valid eNO group (*n* = 273), *n (%)*	*P* value
Male	105 (57)	137 (50)	0.1531
Prematurity	21 (11)	25 (9)	0.4331
Caesarean delivery	67 (36)	91 (33)	0.5475
Exclusive breast feeding at time of test	88 (48)	139 (51)	0.5672
FENO treatment group (maternal)	101 (55)	136 (50)	0.2953
Smoking in pregnancy	19 (10)	28 (10)	> 0.9999
First born	103 (56)	139 (51)	0.2949
**Anthropometric data**	**Median (Min, Max)**	**Median (Min, Max)**	
Gestational age at birth (weeks)	39.1 (33.3, 41.7)	39.0 (27.7, 42.4)	0.6324
Birthweight (kg)	3.36 (1.71, 4.88)	3.40 (0.66, 5.30)	0.4198
Chronological age at test (days)	49 (28, 104)	49 (25, 137)	0.7172
Weight at test (kg)	4.8 (3.1, 7.8)	4.8 (3.2, 7.2)	0.5186
Length at test (cm)	55.5 (49.0, 62.1)	56.0 (39.0, 64.0)	0.1286
**eNO**	**Median (Min, Max)**	**Median (Min, Max)**	
eNO50 ppb	11.8 (0.13, 47.84)	-	
eNO50SqR	3.44 (0.36, 6.92)	-	

*eNO* exhaled nitric oxide, *FENO* Fractional exhaled nitric oxide, *eNO50* exhaled nitric oxide interpolated to expiratory flow rate 50 ml per second, *eNO50SqR* square root eNO50

**Fig. 2:**
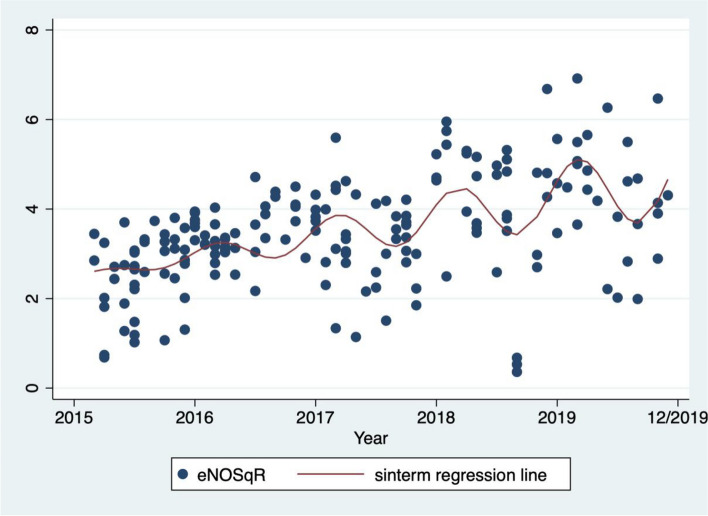
eNO50 measured in each infant (transformed to square root) and plotted by month of test through the duration of the study, with sinterm regression line overlayed. eNO50SqR: square root eNO50 (exhaled nitric oxide interpolated to expiratory flow rate 50ml per second); sinterm: parameterized sine model with assumed value of zero at June, peak in March, and trough in September with increasing amplitude for each year

Variation with seasons was evident, with the median eNO50 (13.6 ppb) in the warmer months (November to March) being significantly greater than the median eNO50 (11.0 ppb) in the cooler months (April to October), *P* = 0.008 as shown in Table 2. Figure 2 (eNOSqR with sinterm regression line) shows the eNOSqR for each infant across the five years of the study with the sinterm function overlayed.

**Table 2:** Median eNO50 by warmer/cooler months

Variable	Warmer months	Cooler months	*P* value
eNO50 (ppb)	13.6	11.0	0.008

*eNO50* exhaled nitric oxide interpolated to expiratory flow rate 50 ml per second, warmer months: November to March (with mean daily temperatures above 20 degrees Celcius); cooler months: April to October (with mean daily temperatures below 20 degrees Celcius)

**Fig. 3:**
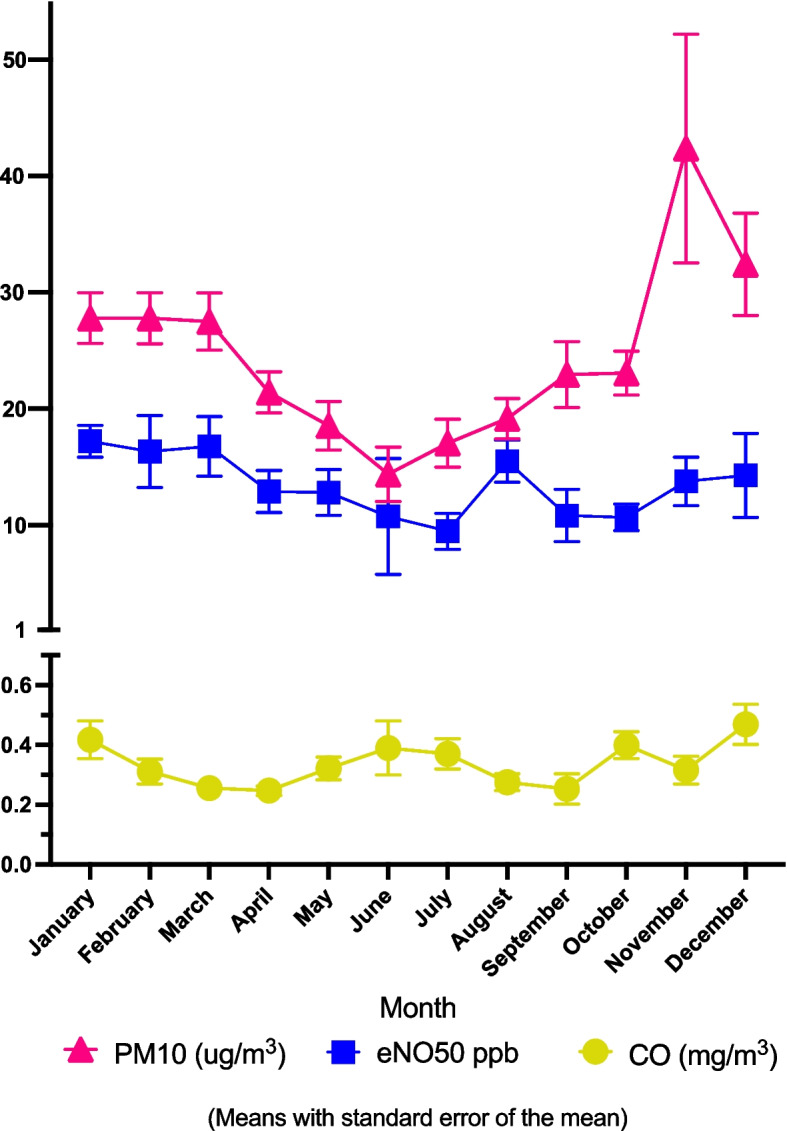
CO: carbon monoxide; eNO50: exhaled nitric oxide interpolated to expiratory flow rate 50ml per second; PM10: particulate matter less than 10μm; SO2: sulfur dioxide

**Fig. 4:**
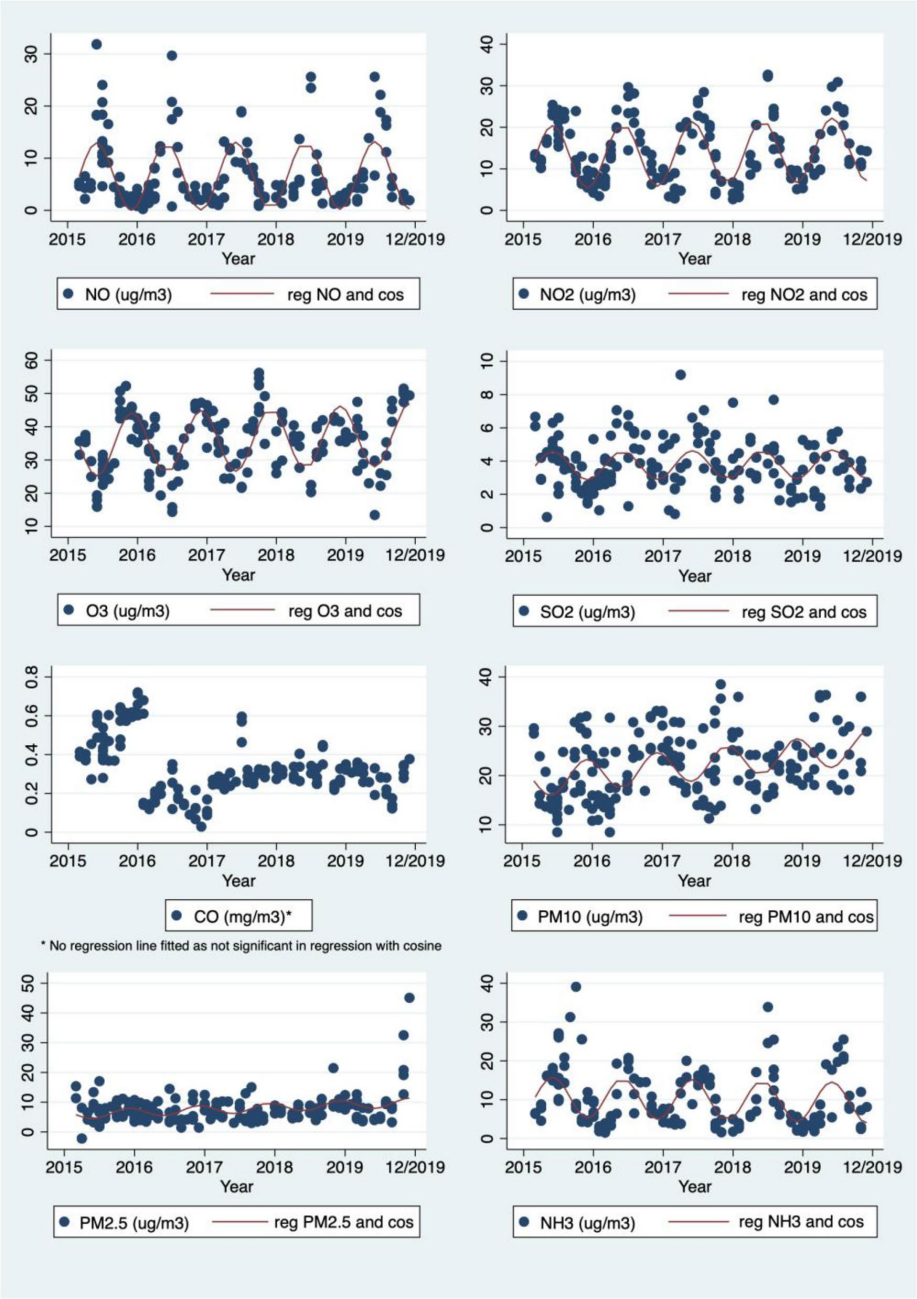
Seven-day mean air pollutants on the day of the infant’s clinical assessment, plotted by month of test through the duration of the study, with cosine regression line overlayed. CO: carbon monoxide; cos: cosine; NH3: ammonia; NO: nitric oxide; NO2: nitrogen dioxide; O3: ozone; PM10: particulate matter less than 10μm; PM2.5: particulate matter less than 2.5μm; reg: regression line; SO2: sulfur dioxide

Weather and air pollution medians and range for the days when infants were clinically assessed are shown in Table 3. The number of measurements for each day vary due to missing data from the Bureau of Meteorology or New South Wales Air Quality Monitoring Network. Air pollutants varied across seasons as seen in Fig. 3 (Mean eNO50, CO and PM10 by month), Fig. 4 (Air pollution with cos regression line for seven-day mean air pollutants) and Supplement Fig. 1 (Mean eNO50, NO, NO_2_, O_3_, PM2.5 and SO_2_ by month). For instance, ozone levels were higher in and around the Australian summer months (December to February), and low around the Australian winter months (June to August) as expected. The following air pollutants had significant associations in linear regression with cosine: 24 h to 12 pm mean NO, NO_2_, O_3_, CO, PM10, PM2.5, NH_3_, and seven-day NO, NO_2_, O_3_, SO_2_, PM10, PM2.5, and NH_3_. (Seven day means with cosine regression lines shown in Fig. 4, Air pollution with cos regression line for seven-day mean air pollution).

**Table 3:** Weather and air pollutants for dates when infants were clinically assessed

Weather	*n*^a^	eNO group Median (Min, Max)
Maximum daily temperature (°C)	147	24.6 (14.0, 41.0)
Mean daily temperature (°C)	155	19.4 (9.3, 30.6)
Mean 24h humidity (%)	156	70.4 (30.7, 98.2)
Mean 9am humidity (%)	152	69.4 (28.7, 100.5)
**Air pollutants 24-h mean**	n^a^	**Median (Min, Max)**
CO mg/m^3^	163	0.30 (0.02, 0.88)
NH_3_ μg/m^3^	163	6.94 (-0.52^b^, 53.69)
NO μg/m^3^	164	3.05 (-0.59^b^, 57.76)
NO_2_ μg/m^3^	164	12.62 (-0.65^b^, 43.92)
O_3_ μg/m^3^	164	34.34 (5.88, 65.87)
PM10 μg/m^3^	166	21.81 (4.75, 169.68)
PM2.5 μg/m^3^	165	7.19 (-2.2^b^, 45.11)
SO_2_ μg/m^3^	163	3.08 (-0.92^b^, 20.05)

^a^There were 166 distinct dates when infants were clinically assessed. *n* refers to the number of distinct dates when infants were clinically assessed that each weather aspect and air pollutant were available due to missing data from the Bureau of Meteorology or New South Wales Air Quality Monitoring Network

^b^Negative values are reported due to the accuracy limitations of the measuring instrument

*CO* Carbon monoxide, *eNO* exhaled nitric oxide, *NH*_*3*_ ammonia, *NO* Nitric oxide, *NO*_*2*_ Nitrogen dioxide, *O*_*3*_ Ozone, *PM10* Particulate matter less than 10 μm, *PM2.5* particulate matter less than 2.5 μm, *SO*_*2*_ Sulfur dioxide

**Fig. 5:**
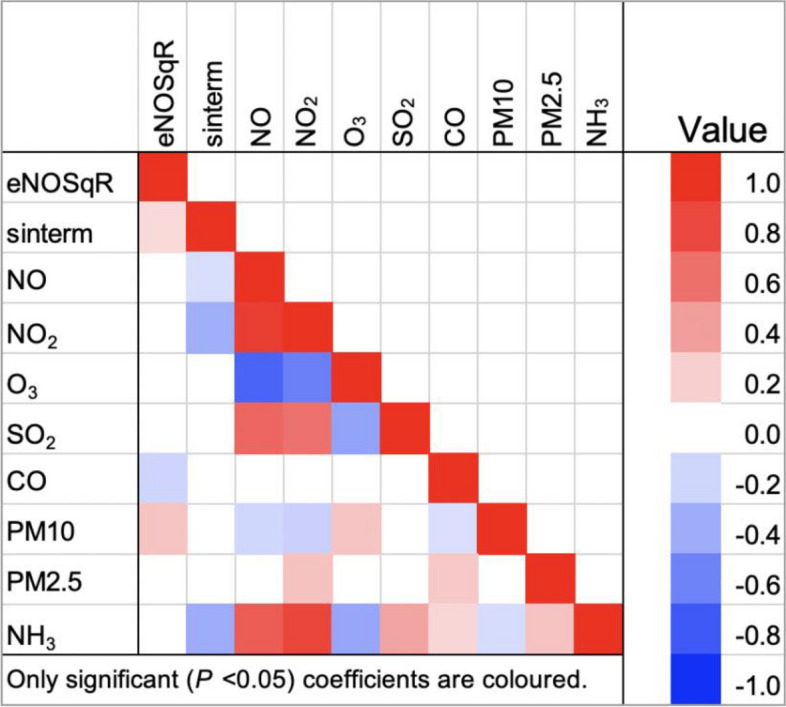
CO: carbon monoxide; eNO50SqR: square root eNO50 (exhaled nitric oxide interpolated to expiratory flow rate 50ml per second); NH3: ammonia; NO: nitric oxide; NO2: nitrogen dioxide; O3: ozone; PM10: particulate matter less than 10μm; PM2.5: particulate matter less than 2.5μm; sinterm: parameterized sine model with assumed value of zero at June, peak in March, and trough in September with increasing amplitude for each year; SO2: sulfur dioxide

eNO showed a weak positive correlation with sinterm and seven-day mean PM10 and weak negative correlation with seven-day mean CO. (Fig. 5, Heatmap of Spearman correlation coefficient for eNO, sinterm and seven-day mean air pollutants). Between pollutants, the strongest positive correlations were between seven-day mean NO and NO_2_. Moderate positive correlations were observed between both seven-day mean NO and NO_2_ with seven-day mean SO_2_ and NH_3_. Strong negative correlations were evident between seven-day mean O_3_ with both seven-day mean NO and seven-day mean NO_2_. Moderate negative correlations were found between seven-day mean O_3_ and seven-day mean NH_3_.

**Fig. 6:**
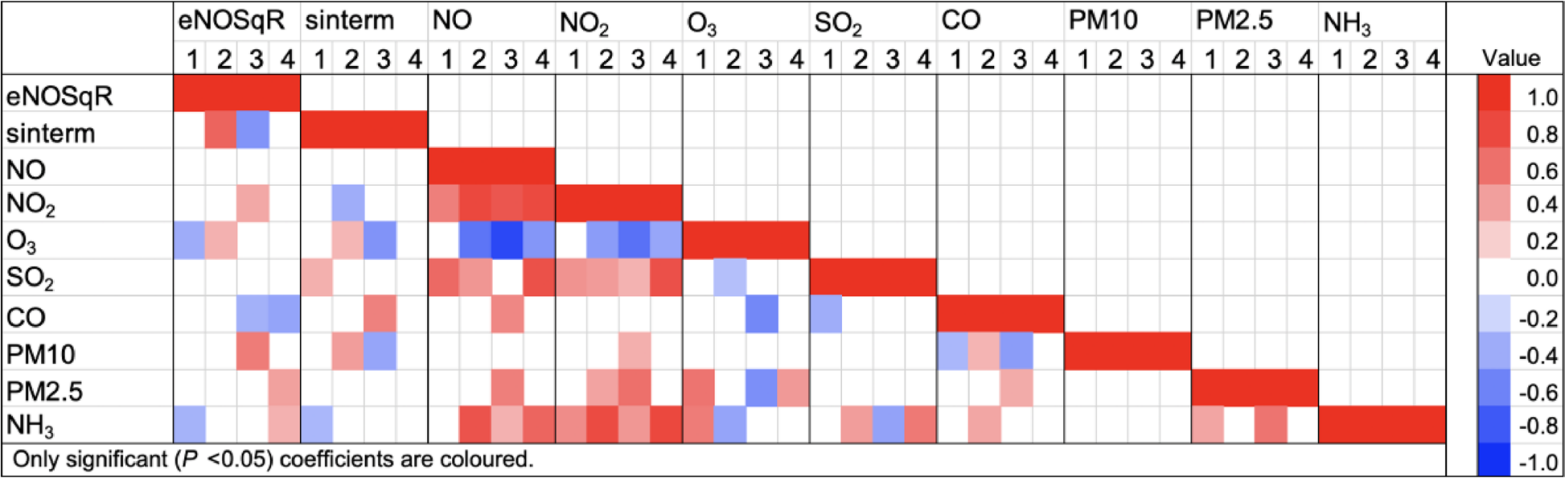
1: Summer; 2: autumn/fall; 3: winter; 4: spring; CO: carbon monoxide; eNO50SqR: square root eNO50 (exhaled nitric oxide interpolated to expiratory flow rate 50ml per second); NH3: ammonia; NO: nitric oxide; NO2: nitrogen dioxide; O3: ozone; PM10: particulate matter less than 10μm; PM2.5: particulate matter less than 2.5μm; sinterm: parameterized sine model with assumed value of zero at June, peak in March, and trough in September with increasing amplitude for each year; SO2: sulfur dioxide

After grouping into seasons, the coefficients showed some variability in their association with eNO relative to overall coefficients (Fig. 6, Heatmap of eNO, sinterm and seven-day mean air pollutants by season). Seven-day mean NO_2_ had a positive association in winter, seven-day mean O_3_ had negative association in summer, but positive association in autumn/fall, seven-day mean PM2.5 had a positive association in spring and seven day mean NH_3_ had a negative association in summer, but positive association in spring.

Adjusted for seven confounders (age at test, birth order, male, birth weight, prematurity, exclusive breastfeeding, maternal smoking in pregnancy), the LASSO linear regression model for eNO identified nine non-zero coefficients: sinterm, seven day mean CO, 24 h mean NH_3_, seven day mean PM10, 24 h mean NO, seven day mean PM2.5, seven day mean O_3_, 24 h mean SO_2_ and seven day mean NH_3_ with an R-square of 0.25 for the penalized coefficients. When the nine non-zero coefficients (and confounders) were entered in multivariable regression the explained variance was 27% (Table 4).

**Table 4:** Multivariable regression of all LASSO active coefficients for eNO

Variable	Coefficient	95% CI	*P* value
sinterm	0.004	0.002, 0.005	< 0.001
Seven-day mean CO	-1.627	-2.742, -0.512	0.005
24-h mean NH_3_	0.017	-0.006, 0.04	0.146
Seven-day mean PM10	0.026	0, 0.052	0.051
24-h mean NO	-0.015	-0.034, 0.004	0.112
Seven-day mean PM2.5	0.041	-0.009, 0.09	0.105
Seven-day mean O_3_	0.016	-0.009, 0.041	0.214
24-h mean SO_2_	-0.004	-0.056, 0.048	0.873
Seven-day mean NH_3_	0.023	-0.016, 0.061	0.248

R^2^ 0.265, *P* value 0.0000

Adjusted for age at test, birth order, infant sex, birth weight, prematurity, exclusive breastfeeding, and maternal smoking in pregnancy

*CO* Carbon monoxide, *eNO* exhaled nitric oxide, *NH*_*3*_ Ammonia, *NO* Nitric oxide, *NO*_*2*_ Nitrogen dioxide, *O*_*3*_ Ozone, *PM10* Particulate matter less than 10 μm, *PM2.5* Particulate matter less than 2.5 μm, *sinterm* parameterized sine model with assumed value of zero at June, peak in March, and trough in September with increasing amplitude for each year; SO_2_: sulfur dioxide

## Discussion

This study is the first to our knowledge to demonstrate an association between eNO in young infants born to mothers with asthma in pregnancy and postnatal air pollution exposure. eNO also showed a significant seasonal variation. Infants born to asthmatic mothers may be more sensitive to the effects of air pollution due to their immature lungs as they are at a higher risk of developing asthma (23) and have lower lung function at 6 weeks of age [34]. We have also demonstrated that the exposure to ambient air pollutants during pregnancy is associated with shifts in cord blood cell types which may cause an inflammatory response in the placenta and potentially influence fetal development [35]. We further investigated that those infants with impaired lung function had increased expression of chemoattractant receptor homologous molecule on cord blood group 2 innate lymphoid cells [36]. Recently, Decrue et al. [37] have shown significant associations of PM10 during the second trimester of pregnancy with lung function and FeNO in term and preterm infants. Preterm infants showed significantly higher susceptibility even to low to moderate prenatal air pollution exposure than term infants, leading to increased impairment of postnatal lung function. Together there is now evidence to suggest that both prenatal [37] and postnatal (this study) PM10 exposure is associated with eNO levels at six weeks of age.

The median eNO in this study was similar to the BILD cohort using a coherent methodology [30], but higher than that reported by a 2021 non-systematic review [32]. However, the infants in our study were older than the infants in the review and were infants of asthmatic mothers, both factors having been associated with higher eNO measurements in infants [30, 38]. While the technical methodology was coherent between our study and the BILD cohort [30], the populations were slightly different as our cohort [34] included infants born prematurely, with higher rates of caesarean section delivery, lower proportion of exclusive breastfeeding at six weeks. In addition, all mothers had asthma in pregnancy and there was no restriction on ethnicity for enrolment in our study [39].

Air pollution has been measured continuously in Newcastle since 1993 [40]. Compared to international standards, air pollution locally is relatively low, with NO_2_, CO, and SO_2_ not exceeding National Environment Protection Measure for Air standards [41] since monitoring began [40]. O_3_, PM10 and PM2.5 periodically exceed the national standards with varying weather, location and intensity of transport and industry emissions, and natural events such as bushfires and dust storms [40]. Newcastle is predominantly urban and has a declining industrial nature. However, Newcastle remains the world’s largest coal and largest tonnage throughput port in Australia, with over 2,200 trade vessels visiting each year [42], with coal particles estimated to account for about 10% of ambient PM10 levels in Newcastle [43]. Furthermore, large bushfires impacted the region during November 2018, November 2019 and December 2019 on four dates when infants attended for eNO measurement and is evident in the marked increase of mean PM10 levels in November and December in Fig. 3. Excluding measurements from those four dates results in mean PM10 in November 26ug/m^3^ and December 29ug/m^3^.

In this study, seasonal variation was observed in both eNO and air pollution concentrations, with mean eNO highest in the warmer months and lowest in the cooler months. The LASSO model identified positive associations for eNO with NH_3_, PM10, PM2.5 and O_3_, and negative associations for eNO with CO, NO and SO_2_. This aligns with other studies in children showing a positive association of eNO with PM2.5, PM10, and O_3_, [20, 21] but contrasts with findings of a Polish study [21], which identified a weak positive association between CO and eNO in school-aged children. A 2020 meta-analysis (including adult and child studies) by Chen et al. [44] reported that an increase in short term exposure (less than 14 days) to PM2.5, PM10, NO_2_ and SO_2_ was associated with increased eNO but no associations were seen between short term O_3_ exposure and eNO.

Our study observed a negative association between ambient CO and eNO. Other studies [45–47] have reported varying relationships between ambient CO and eNO. Zhao et al. [46] also discerned a significant negative association between ambient CO and eNO in a cohort of healthy college students. Zhao assumed that at low ambient levels, CO may have anti-inflammatory effects on the respiratory system possibly via interaction with NOS2, which is supported by the findings of a review from 2013 [48].

### Mechanisms

The exact mechanism by which air pollutants affect eNO concentration is not clear, however, it is hypothesized to be via an inflammatory pathway involving NOS2 expression [49]. O_3_, NO_2_ and PM2.5 have been shown to induce airway inflammation [50, 51] and oxidative stress in the airways [52], which then leads to increased production of NO by NOS2 [21]. The arginase-nitric oxide synthase pathway may also be impacted by the oxidative effect of air pollution on DNA methylation of *NOS2* [53], *NOS3* [54], *ARG1* and *ARG2* [55], resulting in increased eNO production.

### Strengths and limitations of study

The strengths of this study include that it is the largest population of infants born to mothers with asthma in which eNO has been measured at six weeks of age. These infants are at increased risk of asthma in later life and may be more susceptible to air pollutants [56]. Another strength is the duration of the study, with an observation period of almost five years, decreasing the possibility that outlying variations in smaller time periods confounded results. Additionally, the techniques for measuring infant eNO and lung function are derived from those used in the largest longitudinal study of infant lung development – the BILD Cohort [39].

A limitation of this study is that the single location used for measuring air pollution does not provide an exact assessment of the outdoor air pollution at the infant’s home address or household air pollution exposure. However, all infants were living within the local region at the time of their test. Nonetheless, the use of daily regulatory monitoring data along with land-use regression models capable of predicting the spatiotemporal variation in pollutants could further improve the precision of individual exposure estimates.

Another potential limitation is that any potential effect from prenatal air pollution exposure has not been accounted for, whereas other studies have described associations between eNO in infants and prenatal air pollution exposure [18]. Further research is needed to determine if there are differences in the impact on infants between exposure to air pollutants in-utero compared to the neonatal period.

There were issues with equipment failure and lack of access to NO-free air, but we are confident that this did not induce selection bias as it occurred at random.

## Conclusion

In conclusion, eNO in young infants of mothers with asthma in pregnancy was associated with local air pollution concentrations, and seasonal variation of eNO was observed. While the absolute change in median eNO is small (2.6 ppb), the relative change was a 24% increase from cooler months to warmer months. In adults or older children this change would not be clinically significant [57], however, in infants the clinical significance of eNO and any change in eNO is less clear. These findings suggest that additional seasonal factors such as local air pollution concentrations need to be accounted for when measuring eNO in young infants of asthmatic mothers, and that even low levels of ambient air pollution may modulate nitric oxide production in the airways of infants.

### Supplementary Information

**Additional file 1.**

## Data Availability

The data generated in this study are not publicly available due to privacy or ethical restrictions but are available from the corresponding author on reasonable request.

## Abbreviations

ATS: American Thoracic Society
BILD: Bern Basel Infant Lung Development
BLT: Breathing for Life Trial
CO: Carbon monoxide
CV: Coefficient of variance
eNO: Exhaled nitric oxide
eNO50: Exhaled nitric oxide at flow rate 50 mL/s
eNOSqR: Square root of eNO50
ERS: European Respiratory Society
LASSO: Least absolute shrinkage and selection operator
NH_3_: Ammonia
NO: Nitric oxide
NO_2_: Nitrogen dioxide
NOS: Nitric oxide synthase
NSW: New South Wales
O_3_: Ozone
PM10: Particulate matter < 10 μm in diameter
PM2.5: Particulate matter < 2.5 μm in diameter
PTE: Prenatal tobacco exposure
SO_2_: Sulfur dioxide
