# Supplementary material for: The association of exhaled nitric oxide with air pollutants in young infants of asthmatic mothers
Source: Environ Health. 2023 Dec 5;22:84. doi: 10.1186/s12940-023-01030-6 (PMC10696885; doi:10.1186/s12940-023-01030-6)
Supplement: Supplementary file 1 — Additional file 1. [file 12940_2023_1030_MOESM1_ESM.pdf]

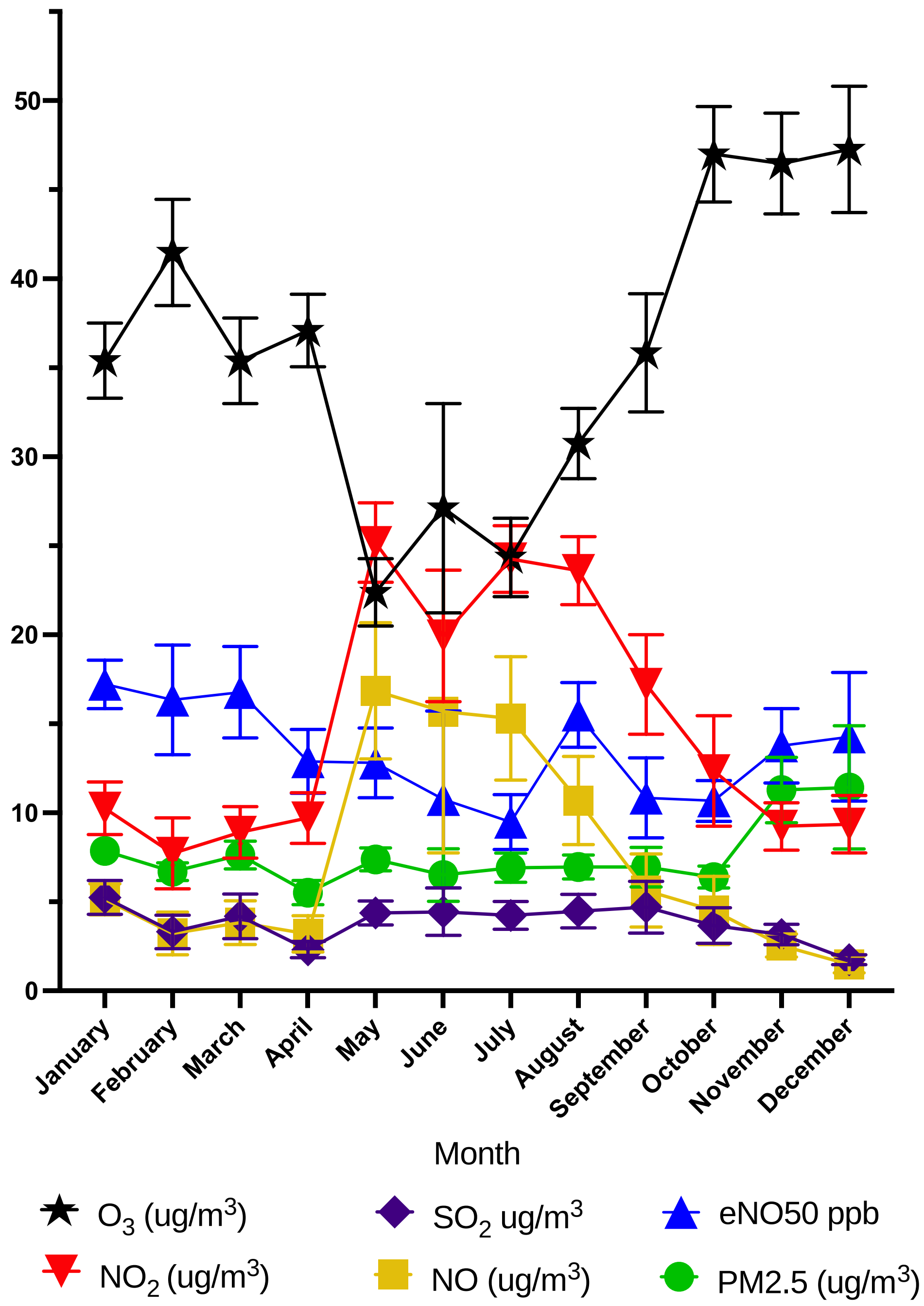

(Means with standard error of the mean)

eNO50: exhaled nitric oxide interpolated to expiratory flow rate 50ml per second; NO: Nitric Oxide; NO_2: Nitrogen dioxide; O_3: ozone; PM2.5: particulate matter less than 2.5µm; SO_2: sulfur dioxide.
